# Effect of Fortification with Fish (*Pseudophycis bachus*) Powder on Nutritional Quality of Durum Wheat Pasta

**DOI:** 10.3390/foods7040062

**Published:** 2018-04-17

**Authors:** Ajay S. Desai, Margaret A. Brennan, Charles S. Brennan

**Affiliations:** 1 Department of Wine, Food and Molecular Biosciences, Lincoln University, Christchurch 7647, New Zealand; ajay.desai@lincolnuni.ac.nz (A.S.D.); margaret.brennan@lincoln.ac.nz (M.A.B.); 2 Riddet Research Institute, Palmerston North 4442, New Zealand

**Keywords:** Semolina pasta, fish powder, fortification, nutrients digestibility, antioxidant activity

## Abstract

This paper investigates the nutraceutical (phenolic content and antioxidant activity) and nutritional potential (protein and starch digestibility) of supplementation of durum wheat semolina with 5–20% fish powder (*Pseudophycis bachus*). In general, all enriched pasta with fish powder showed a significant decrease (*p* < 0.05) in reducing sugar released during an in vitro digestion, and reductions in standardized area under the curve (AUC) values, compared to control pasta. The potentially bioaccessible fraction of pasta enriched with 20% fish powder (FP) was characterized as having a 177–191% increase in phenolic content and a 145–556% higher antiradical activity. Elevation of these parameters in fortified pasta was accompanied by interaction of wheat starch, protein, and fish powder protein. Supplementation of fish powder also influenced protein digestibility (a reduction from 84.60% for control pasta to 80.80% for pasta with 20% fish powder). Fortification improved the nutraceutical and nutritional potential of the studied pasta with the effects depending on factors including protein-starch-phenolic interactions.

## 1. Introduction

There is an increasing trend for consumers to demand the development of nutritionally rich foods while also moderating the quantity of digestible starch due to health concerns related to their high intake. A high intake of readily digestible starch results in increased blood glucose levels and may be related to obesity and being overweight [1]. The worldwide prevalence of diabetes is predicted to increase from 382 million people to 592 million by the end of 2035, and approximately 10% of the population will have diabetes [2]. The search for health enhancing food ingredients for pasta preparation has been growing. A promising approach of examining the enrichment of food ingredients and their physiological effects is by the concept of glycemic index (GI), which is used to predict postprandial blood glucose level [1,3]. Consumption of foods with a low glycemic value could manipulate the effects of diabetes, cardiovascular, and neurodegenerative disease [4]. Low glycemic index foods can be achieved with the utilization of protein-rich and fiber-rich ingredients combined with cereal grains in products such as bread and pasta [5,6]. Pasta is a staple food, containing carbohydrates (74–77%) and protein (11–15%), although pasta is deficient in lysine and methionine. Pasta may be fortified with functional ingredients to alter its nutritional quality, such as with essential amino acids, minerals, vitamin, and phenolic compounds [5]. To achieve this, pasta products have been fortified with high protein sources, such as soya flours, soy isolates, milk and milk products, whey proteins, yeast protein concentrates, and meat [7,8]. Fortification of food is a convenient method and includes the addition of one or more functional components for the purpose of enhancing a biological activity of newly designed food products [9]. In recent years, pasta has been fortified using different ingredients including quinoa flour [10], lentil flour [10], beef meat [11], and freeze dried shrimp powder [12]. The Food and Drug Administration (FDA) and World Health Organization (WHO) consider pasta a vehicle for the addition of nutrients to the diet, as it can be enriched with protein and various bioactive ingredients such as phenolic compounds and dietary fiber [13]. Phenolic compounds exhibit biological properties, such as antioxidant activity. Food rich in polyphenols have the potential to protect against various diseases associated with oxidative damage, such as cardiovascular, cancer, and neurological disease [14].

Fish is not only an excellent source of high value protein but also an important source of essential amino acids and omega-3 (*n*-3) fatty acids, especially eicosapentaenoic acid (EPA) and docosahexaenoic acid (DHA), as well, it contains micronutrients such as vitamins (A, D, B6, and B12) and minerals (iron, zinc, iodine, selenium, potassium, and sodium). The American Heart Association (AHA) recommends a minimum consumption of two fish serving per week (200 mg/day of long chain *n*-3 polyunsaturated fatty acid (PUFA)) to achieve a cardio protective effect. To fulfil these requirements, pasta could be fortified with fish powder. Fish powder is protein-rich and contributes to a low glycemic index and has the potential to have beneficial health effects such as manipulation of obesity, hypertension, and cardiovascular disease [15]. Previous studies have reported the nutritional and physicochemical characteristics of pasta manufactured with fish powder of green mussel *(Perna canaliculus)* [16], shrimp meat (*Penaeus monodon*) [12], and beef meat [11]. However, the nutritional properties of pasta enriched with partial replacement of semolina wheat flour by red cod powder *(Pseudophycis bachus)* is still unknown. The present study was conducted to evaluate the effects of the addition of different levels of fish protein powder on the pasta characteristics including in vitro starch and protein digestibility and antioxidant activity.

## 2. Materials and Methods

### 2.1. Materials

Semolina (Sun Valley Foods, Christchurch, New Zealand) was obtained from Foodstuffs, New Zealand (Christchurch, New Zealand). Red cod *(Pseudophycis bachus)* fish were bought in ice condition from Christchurch Wholesale Seafood (Christchurch, New Zealand). Folin-Ciocalteau reagent, Fluorescein, 2,2′-azobis (2-amidino-propane) dihydrochloride (AAPH), 6-hydroxy-2,5,7,8-tetramethylchroman-2-carbixylic acid (Trolox), pepsin, pancreatin, trypsin, α-chymotrypsin, peptidase, Amyloglucosidase, and 3.5-dinitrosalicylic acid (DNSA) were purchased from Sigma-Aldrich (St. Louis, MO, USA). 

### 2.2. Fish Powder Preparation 

The fish were de-scaled, beheaded, eviscerated, and washed with potable water. The dressed fish were cooked by boiling in water for 10 min. The cooked fish were deskinned and deboned manually before drying in a cabinet dryer (Moffat, E32M, Christchurch, New Zealand) at 45 °C for 16 h. The dried fish muscle was ground to produce a powder (Sunbeam, Model: EM0405, Auckland, New Zealand) and sieved to pass through the appropriate 0.5 mm mesh screen [17]. Dried powder was put in a sealed polythene bag and stored at −20 °C temperature until required.

### 2.3. Pasta Preparation 

Fresh pasta was prepared using an automated pasta extruder with 2.25 mm diameter die with 20 holes (Model: MPF15N235M; Firmer, Ravenna, Italy). Each blend (500 g) was mixed for 5 min in order to ensure a uniform mixture of semolina and fish powder. The conditions applied were the following: tap water temperature 41 °C, dough moisture content 32.5 g/100 g, and mixing time 20 min according to the manufactures guidelines. Extruded fresh pasta samples (20 g) were put into a resealable polythene bag and frozen at −18 °C until required. Prior to analysis, the pasta was defrosted for 10 min at room temperature. Five pasta formulations were prepared in the ratios (semolina/fish powder) of 100:0; 90:5; 90:10; 85:15; and 80:20.

### 2.4. Amino Acid Profile of Semolina and Fish Powder

To hydrolyze the sample protein into its constituent amino acids, the sample was acid digested with 6 N hydrochloric acid in an oven at 110 °C for 20 h. The amino acids in the samples were then determined using an Agilent 1100 series (Agilent Technologies, Walbronn, Germany) high-performance liquid chromatography following the methodology proposed by [18]. The extracted amino acid samples were injected into HPLC equipped with a 150 × 4.6 mm, C18, 3u ACE-111-1546, (Winlab, Scotland, UK) column for amino acid separation. Column flow rate was 0.7 mL/min and the temperature was kept at 40 °C. *O*-phthaldialdehyde (OPA) was used as a fluorescence derivative reagent for primary amino acids, and 9-fluorenylmethyl chloroformate (FMOC) for secondary amino acids. Detection utilized a fluorescence detector with an excitation of 335 nm and emission of 440 nm for primary amino acids. At 22 min, the detector was switched to excitation 260 nm and emission at 315 nm to detect secondary amino acids such as proline. The amino acid results are expressed in milligram amino acids per g protein of sample.

### 2.5. Gastric Digestibility Determination Using In Vitro Starch Digestion Process 

Pasta was cooked in boiling tap water (600 mL) for optimum cooking time (5–6.30 min), cut into 2–5 pieces, and the potential amount of glucose released over 120 min was measured as described by [19]. In brief: digestions were carried out in 60 mL plastic digestion pots placed on a pre-heated 15-place magnetic heated stirring block (IKAAG RT 15, IKA-WERKE Gmbit & Co., Staufen, Germany). Pasta (3.5 g) was mixed with 30 mL of distilled water and placed on a heated stirrer at 37 °C with constant stirring. Stomach digestion was initiated by adding 0.8 mL 1 M HCL and 1 mL of 10% pepsin solution in 0.05 M HCl with continued stirring, and heat was maintained at 37 °C for 30 min. Stomach digestion was stopped by the addition of 2 mL of 1 M NaHCO_3_. Small intestine digestion was mimicked by the addition of 5 mL of 0.1 M sodium maleate buffer pH 6 and 5 mL of 2.5% pancreatin solution in 0.1 M sodium maleate buffer pH 6 followed by the volume being make up to 53 mL with constant stirring at 37 °C for 120 min. Amyloglucosidase (0.1 mL) was added to the digestion pot in order to prevent end product inhibition of pancreatic α-amylase. Aliquots (1 mL) were withdrawn after 0, 20, 60, and 120 min, to which 4 mL ethanol was added to stop any further reaction. The samples were stored at 4 °C until analysis of reducing sugar content using the 3.5-dinitrosalicylic acid (DNS) method by [3] was completed. Glucose release was plotted against time, and area under the curve (AUC) was calculated by dividing the graph into trapezoids as described elsewhere [3]. The in vitro digestion analysis was used to determine predicated glycemic response.

### 2.6. In Vitro Protein Digestibility

The muli-enzyme technique described by [20] was used for the determination of in vitro protein digestibity of cooked pasta samples. A 50 mL protein suspension was prepared in distilled water (6.25 mg of protein/mL), adjusted to pH 8 with a solution of 0.1 N HCL and/or 0.1 N NaOH, and placed on a magnetic heating stirring block at 37 °C. The multi-enzyme solution (1.6 mg/mL trypsin, 3.1 mg/mL chymotrypsin, and 1.3 mg/mL peptidase) was maintained in an ice bath and adjusted to pH 8.0 with 0.1 N HCL and/or 0.1 N NaOH. Five mL of the multi-enzyme solution was then added to the protein suspension, which was maintained at 37 °C. The decrease in pH was measured after the addition of an enzymatic solution at every minute for a period of 10 min using a digital pH meter (S20 Seven Easy^TM^, Mettler Toledo, Columbia, MD, USA). The percent protein digestibility (Y) was calculated by using Equation (1) [20]:

Y = 210.46 − 18.10 X,(1)

where X represents the change in pH after 10 min.

Protein availability refers to the quantity of protein digested in pasta and was calculated as:

Protein availability (PA)=(Protein digestibility × Protein content in cooked pasta)/100

### 2.7. In Vitro Gastro-Intestinal Digestion

As per the method described in Section 2.5, after gastric and pancreatic digestion, aliquots (1 mL) were withdrawn after 30 and 120 min, to which 1 mL ethanol was added (1:1) to stop any further reaction. Thereafter, samples were centrifuged at 1000 rpm for 5 min. Supernatants (gastrointestinal digested extracts) and pellets were separated and kept at −20 °C for further analysis.

### 2.8. Total Phenolic Content

Total phenolic content of supernatant obtained from in vitro gastro-intestinal digestion was measured using the Folin-Ciocalteu method as described by [21]. The Folin-Ciocalteu reagent was diluted to make 0.2 N, and 0.5 mL sample extract was mixed with 2.5 mL of diluted Folin-Ciocalteu reagent (0.2 N). After 3 min, 0.2 mL of 7.5% sodium carbonate solution was added, the contents were mixed, and kept in darkness at room temperature for 2 h. The absorbance was then measured at 760 nm using a spectrophotometer V-1200 model (Schimadzu, Columbia, MD, USA). Gallic acid (0–0.2 mg/mL), prepared in methanol, was used as a standard, and the results were expressed as mg of gallic acid equivalents (GAE)/g sample.

### 2.9. Oxygen Radical Absorbance Capacity (ORAC) Assay

#### 2.9.1. Reagents and Standard Preparation

AAPH (2, 2′azobist (2-amidino-propane) dihydrochloride) (0.645 g) was dissolved in 10 mL of 75 mM phosphate buffer (pH 7.4) and was kept in an ice bath. Fluorescein stock solution (1 mM) was prepared with 0.016 g fluorescein dissolved in 50 mL of 75 mM phosphate buffer (pH 7.4) and was kept at 4 °C in dark condition. A 10 nM fresh fluorescein working solution was made daily by further diluting the stock solution in 100 mL phosphate buffer (pH7.4). Trolox (6-hydroxy-2,5,7,8-tetra methylchroman-2-carboxylic acid) standard was prepared as follows: 0.0250 g of Trolox was dissolved in 50 mL of 75 mM phosphate buffer (pH 7.4) to obtain a 2 mM stock solution. The stock solution of Trolox was diluted with phosphate buffer to 100 μM, 50 μM, 25 μM, 12.5 μM, 6.25 μM, 3.125 μM, 1.5625 μM, and 0 μM working solutions.

#### 2.9.2. Oxygen Radical Absorbance Capacity (ORAC) Assay

The ORAC assay was used in this study according to [22]. A 96-well microplate reader (FLUOstar Omega, BMG LABTECH, Ortenberg, Germany) was used with fluorescence filters for an excitation wavelength of 544 nm and an emission wavelength of 590 nm, bottom reading with 50 flashes per well. The plate reader was monitored by BMG LABTECH’s MARS Data Analysis (3.02 R2 version) Software (BMG LABTECH, Ortenberg, Germany). Dilution of sample and standards was done manually. The quantity of 150 μL phosphate buffer pH (7.0) was added to the outer well of the 96-well flat bottom polystyrene microplate (Corning Incorporated, Corning, NY, USA) by hand pipet as well as 25 μL Trolox standard dilutions to the standard 96-well plate reader, followed by 25 μL of sample of different dilution. Finally, 150 μL of fluorescein (10 nM) was added to each well including Trolox and the sample well but excluding the outer well. The plate was covered with an adhesive sealing film, incubated in a microplate for 30 min at 37 °C. After incubation, the adhesive film was removed from the plate reader and 25 μL of AAPH solution was added to each well containing Trolox and the sample. This had to be done as quickly as possible since the reactive oxygen species (ROS)-generator displays immediate activity after addition. The test was resumed and fluorescent measurements were taken up to 60 min.

### 2.10. Statistical Analysis

All experiments were performed in triplicate unless otherwise stated. Data obtained during the study were subjected to one-way analysis of variance (ANOVA), and significance difference in the response and sample were evaluated by Tukey’s comparison test (*p* < 0.05). Statistical software version 16 (Minitab, Sydney, Australia) was used to perform the statistical analysis of the data.

## 3. Results and Discussion

### 3.1. Amino Acid Profile of Semolina and Fish Powder

The amino acid profile of wheat semolina and fish powder samples are presented in Table 1 as compared to the bovine serum albumin protein standard. The amino acids of fish powder were higher than that of semolina. The ratio of the total essential amino acids to the total amino acids (TEAA/TAA) was higher in fish powder than in the semolina. The essential amino acid content of the semolina and fish powder was compared with the recommendations made by Food and Agriculture Organisation/World Health Organisation/United Nations University (FAO/WHO/UNU) (2007) [23] for adult humans. Fish powder samples exceeded the essential amino acid requirements for adult human and infants while the essential amino acid of semolina was below the adult and infant requirements. Sathivel et al. [24] reported that herring fish powder exceeded the essential amino acid requirements for adult humans.

### 3.2. In Vitro Predictive Glycaemic Response

**Figure 1:**
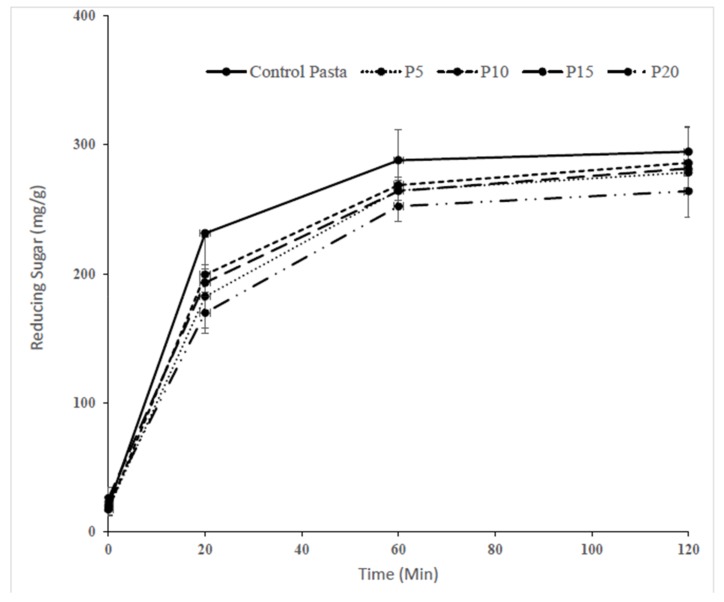
Amount of reducing sugar released during in vitro *d*igestion. CP-control pasta, P5-P20 pasta fortified with 5–20% fish powder, respectively.

An in vitro enzymatic digestion was performed to evaluate the nutritional quality of the pasta enriched with fish powder, in terms of its starch digestibility and predictive glycemic response. The addition of fish powder into pasta decreased (*p* < 0.05) the extent of in vitro starch digestion compared to the control pasta (Figure 1). The pasta enriched with 20% fish powder exhibited significantly the lower values (*p* < 0.05) of reducing sugar followed by the 5%, 15%, and 10% fish powder pasta samples, while the control pasta showed higher values at each time point during the digestion. The amount of rapidly digestible starch (RDS) in pasta enriched with fish powder was lower than the control (Table 2). Chillo et al. [13] reported that addition of protein-rich soya bean flour into spaghetti significantly lowered the RDS fraction when compared to semolina spaghetti. Also, Brennan et al. [3] found a similar result in mushroom enriched extruded product, which showed that mushroom incorporation restricted the RDS from the fiber enriched products. Table 2 shows the RDS value and area under the curve in control and pasta enriched with fish powder.

Thus, the in vitro digestion of pasta fortified with fish powder demonstrated that the rate and extent of reducing sugar release decreased. This may be due to the incorporation of protein-rich ingredients into pasta that could modify the integrity of the protein network. Several researchers have studied the effects of the addition of protein-rich ingredients into pasta on starch digestibility [12,25]. The protein content of enriched pasta with fish powder increased proportionally to the increasing levels of fish powder added based on the original content of fish powder (88.54%). The addition of fish powder may create a protein network around the starch molecules and reduce the starch granules’ surface accessibility of α-amylase to starch and, hence, affect the enzyme’s ability to hydrolyze the starch into reducing sugar. Similar results were reported by [8] who found that inclusion of 15%, 30%, and 45% beef meat into pasta exhibited a significant decrease in reducing sugar release. Also, Ramya et. al [12], who studied the in vitro starch hydrolysis of pasta made with semolina fortified with different levels (2.5%, 5%, and 10%) of shrimp (*Penaeus monodon*) meat, showed a significant reduction in the reducing sugar release as the concentration of shrimp meat increased. Hager et al. [26] studied the effects of the addition of oat flour on egg pasta formulation. Pasta enriched oat flour exhibited significantly lower reducing sugar compared to control and predicated the glycemic index at different time points. This may be due the higher addition level of egg white powder. It was previously reported that the presence of protein in the food matrix creates a stronger network and reduces the capacity of enzymes to attack the starch granules, thereby delaying starch digestion [13]. Rodriguez De Marco et al. [25] observed a similar effect on starch digestibility with the addition of spirulina biomass in wheat bread pasta. They observed that spirulina formed a protein matrix as a physical barrier around the starch granules and protected them from enzyme attack. The protein-rich fish powder used in this study may also decrease the reducing sugar release due to the formation of a protein network which entraps the α-amylase. Table 2 illustrates the effects of substituting semolina flour with fish powder on standardized AUC values compared to the control pasta sample. The AUC values were decreased in pasta fortified with fish powder as compared to control. These results illustrate, again, that the fish powder enriched pasta was more resistant to starch digestion compared to the control pasta without fish powder. The addition of fish powder into pasta-like products is a convenient and novel choice in lowering the glycemic index of the final product.

### 3.3. Protein Content and In Vitro Protein Digestibility

**Figure 2:**
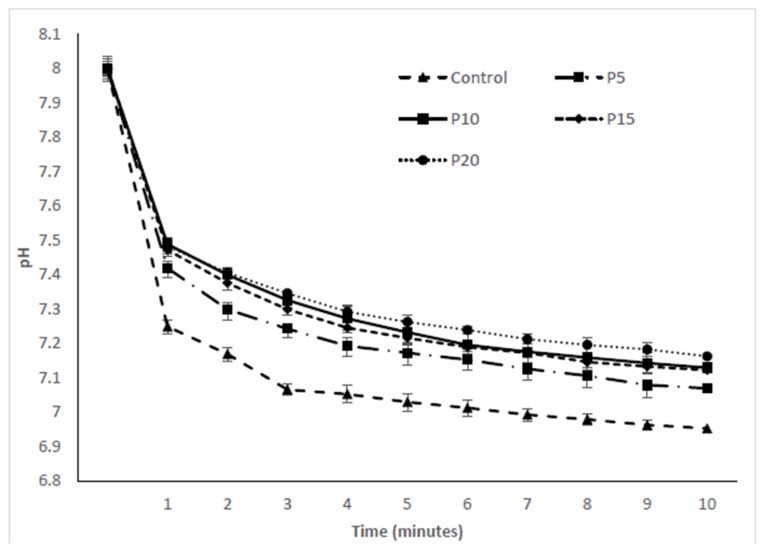
The pH vs. time curves obtained by pasta made with different concentrations of fish powder incubated with multi-enzymes (trypsin, chymotrypsin, and protease).

Protein quality is one of the most important attributes to determine the nutritional characteristics of a food matrix and is evaluated by protein digestibility [9]. Table 3 shows the values of in vitro protein digestibility, protein content in cooked and uncooked pasta, and protein availability after digestion. The authors were unable to find previous information about the in vitro digestibility of pasta enriched with red cod fish powder. The addition of fish powder resulted in a significant (*p* < 0.05) increase in protein content of pasta samples, however, no significant difference (*p* > 0.05) was observed between uncooked and cooked pasta, indicating that during the cooking process, protein did not leach out. This result is in agreement with [25] who found that pasta fortified with spirulina biomass increased the protein content. Fish protein is popularly considered to have high digestibility due to the lack of strong collagenous fiber and tendons which facilitates its use for human consumption [27]. However, in this study, the percentage of in vitro protein digestibility of pasta enriched with fish powder was significantly (*p* < 0.05) reduced (84.60 to 80.80%) as a result of the increase in the level of the fish powder. Similarly, pasta which was made with freeze dried shrimp meat replacement was shown to have a lower in vitro protein digestibility (72 to 90%) than a control (93%) [12]. Rodriguez De Marco et al. [25] also reported that in vitro protein digestibility of spirulina enriched pasta decreased significantly (80.88 to 55.45%) as the incorporation level increased (5 to 20%), proposing that the reduction in digestibility was due to the phenolic compounds present in the spirulina. Protein digestibility in the food matrix depends on factors such as polysaccharides and phenolic molecules. The interaction of phenolic compounds with protein may lead to changes in the digestibility. It has been proposed that oxidized phenolic compounds may react with proteins and form insoluble complexes, inhibiting the activity of proteolytic enzymes and interfering with utilization of proteins [28]. The pH drop curves obtained from enriched pasta with fish powder by using a three enzyme (trypsin, α-chymotrypsin, and protease) system are shown in Figure 2. The drop in pH results from the release of amino acids and peptides, protein building units, as protein is digested. After the addition of multi-enzymes to the protein solution, carboxyl (−COO^−^) and amino (−NH_3_^+^) groups are released. At neutral pH (8.0), the free amino groups deionize in water and protons (H^+^) are liberated. The free H+ released into the surrounding reaction medium causes as decrease in pH. At alkaline pH, phenolic compounds present in the enriched pasta could be oxidized by oxygen with side amino groups of peptides to form quinines, and this lead to the formation of protein cross-links. These quinines react with sulfhydryl and amino groups of proteins and result in decreased protein digestibility [29].

### 3.4. Phenolic Content and Antioxidant Activity

From the consumers’ point of view, after product palatability, the most important factor is bio-accessibility of food components. In the present study, the in vitro digestion process showed fortified pasta with fish powder releases a significant amount of antioxidants. Fish protein have bioactive properties, but they are not so extensively studied as the peptides from other sources such as milk [30]. In general, consumption of fish has several beneficial health effects because of the high content of easily digestible bioactive peptides [31]. The results describing the effect of pasta fortification on its phenolic content and antioxidant activity are presented in Table 4. Phenolic content and antioxidant activity were positively associated with the percentage of fish powder addition, and the highest values were obtained for pasta fortified with a 20% supplement. In comparison to the control, after gastric and pancreatic digestion, the amount of bio-accessible total phenolic compounds in fortified pasta was significantly (*p* < 0.05) higher, with an increase of 1.92 to 2.77 mg of gallic acid/g of pasta for gastric digestion (representing an increase of 123 to 177%) and 2.73 to 5.23 mg of gallic acid/g of pasta for pancreatic digestion (an increase of 122 to 191%). This indicates that adding fish powder ingredients is an alternative to enhance the phenolic content of pasta. Antioxidant activity was observed by the oxygen radical absorbance capacity (ORAC) mechanism. An elevation from 4.39 to 24.45 μmol Trolox/g of pasta (gastric digestion) and 68.97 to 99.31 μmol Trolox/g of pasta (pancreatic digestion) by supplemented pasta (5–20%) was observed. The bio-accessibility of phenolic compounds after digestion varied according to the enriched pasta with fish powder. The total phenolic content and antioxidant activity of the control sample was lower (1.56 mg of gallic acid/g of pasta) than the fortified pasta. This could be due to leaching of phenolic compounds into the cooking medium with higher cooking time. However, retention of phenolic content was significantly higher in fish powder containing pasta as compared to that of the control samples (*p* < 0.05). This clearly indicates that incorporation of fish powder results in the retention of phenolic compounds in the pasta upon cooking. However, [32] observed a significant decrease in total phenolic content in cooked sorghum fortified pasta compared to raw formulations, as also observed by [33] in seaweed enriched pasta. Both researchers were in agreement that during cooking, phenolic compounds leached into the cooking medium and degraded due to thermal treatment. The loss of antioxidant activity due to cooking processes has been reported in another investigation [34], which suggested that during cooking, there is more leaching of bioactive compounds from pasta with durum wheat semolina [35]. In the present study, the pasta fortified with fish powder was able to retain phenolic compounds upon cooking. Similar positive correlations between phenolic level, antioxidant activity, and the supplemental level of fortified pasta have been recorded previously [16,25,34,36,37]. For instance, results obtained by [16] show an increase in ability to reduce by 132% the DPPH (1,1-dephenyl-2-picrylhydrazyl) radical activity of cooked pasta enriched with 5% green mussel powder. On the other hand, Ozdal et al. [38] reported that protein and phenol interact with each other through covalent or non-covalent interactions. These interactions might lead to the precipitation of protein from the food matrix, with studies from [39] showing that covalent bonding may affect both the secondary and tertiary structure of protein. Besides the type and level of functional ingredients used, other factors such as processing and cooking are responsible for alteration of antioxidant properties of pasta [40]. Additionally, the presence of oxygen, water, and heat treatment during cooking and pasta making may induce the oxidation of sensitive phenolic antioxidants [32].

## 4. Conclusions

This study demonstrated that the addition of fish powder in pasta is an effective method to enhance essential amino acid content, protein content, starch digestibility, and the antioxidant potential. This may be beneficial to prevent the outbreak of chronic diseases related to oxidative stress, such as type 2 diabetes, and for improved intestinal health. The significant reduction in glucose release during in vitro digestion of pasta fortified with fish powder compared to the control indicates that there is potential for using protein-rich fish powder in pasta making. Moreover, antioxidant activity from supplemented pasta are highly bio-accessible in vitro. However, the quality of fortified pasta is affected by multiple factors, including protein-starch and protein-phenol interactions. These interactions between fish powder protein and durum wheat starch affect protein-influenced antioxidant activity and starch and protein digestibility of fortified pasta. In addition, further work is required to evaluate the consumer acceptability of pasta fortified with fish powder. In summary, to develop a pasta-like product with protein-rich ingredients, knowledge of the interaction between protein, starch and phenolic in the food matrix is necessary.

## Figures and Tables

**Table 1:** Amino acid profile of semolina, cod fish powder, and bovine serum albumin ^a^.

Amino Acid	S	CFP	BSA	EAA ^b^
*Essential Amino Acids (EAA)*				
Methionine	4.57 ± 3.82	53.32 ± 3.27	15.96 ± 1.43	16
Phenylalanine	43.96 ± 3.36	65.43 ± 3.30	105.38 ± 6.60	38
Isoleucine	28.81 ± 2.71	78.81 ± 3.69	40.36 ± 2.96	30
Lysine	19.88 ± 1.49	202.83 ± 7.23	276.28 ± 7.57	45
Leucine	69.76 ± 7.22	185.35 ± 8.77	263.55 ± 6.52	59
Histidine	21.95 ± 1.68	40.06 ± 2.58	79.70 ± 5.50	15
Threonine	26.65 ± 2.12	84.93 ± 3.05	106.72 ± 6.79	23
Valine	35.55 ± 2.79	76.67 ± 3.34	96.76 ± 6.17	39
*Non-essential amino acids*				
Aspartic acid	23.30 ± 6.03	221.53 ± 6.80	230.83 ± 10.73	
Glutamic Acid	433.18 ± 10.36	364.63 ± 12.32	389.33 ± 16.58	
Cysteine	10.73 ± 3.95	15.92 ± 3.15	130.21 ± 6.56	
Serine	52.06 ± 5.58	99.85 ± 5.18	88.43 ± 6.44	
Glutamine	-	1.24 ± 0.05	1.15 ± 0.01	
Glycine	36.12 ± 2.97	76.89 ± 3.14	28.97 ± 1.69	
Argine	31.40 ± 2.68	116.97 ± 5.13	115.53 ± 7.91	
Alanine	22.64 ± 3.18	129.43 ± 5.71	134.32 ± 9.19	
Taurine	-	3.23 ± 0.17	-	
Proline	90.22 ± 5.74	48.24 ± 9.10	68.15 ± 5.97	
Tyrosine	17.76 ± 1.62	59.78 ± 3.48	89.65 ± 6.00	
TEAA/TAA (%)	25.56 ± 0.50	40.89 ± 0.13	43.56 ± 0.08	

^a^ Data are expressed as mg of amino acid per g of protein. Tryptophan was not determined. S: Semolina, CFP: cod fish powder, BSA: bovine serum albumin, TEAA/TAA: Total essential amino acids/Total amino acids, EAA: Essential amino acid; ^b^ Suggested profile of essential amino acid requirements for adult humans by FAO/WHO/UNU (2007).

**Table 2:** In vitro starch digestibility profile of control and pasta containing fish powder.

Samples	RDS (mg/g Sample)	Area RDS (mg/g Sample)	Total AUC (mg/g Sample)
CP	208.26 ± 1.77 ^a^	56.58 ± 0.23 ^a^	227.87 ± 13.13 ^a^
P5	164.15 ± 21.92 ^a,b^	45.0 ± 1.72 ^ab^	204.23 ± 6.78 ^b^
P10	179.39 ± 26.14 ^a,b^	48.75 ± 2.52 ^ab^	211.32 ± 7.64 ^a,b^
P15	173.84 ± 9.31 ^a,b^	49.30 ± 1.15 ^ab^	207.86 ± 0.83 ^a,b^
P20	152.83 ± 14.61 ^b^	43.35 ± 1.14 ^b^	193.99 ± 7.47 ^b^

Mean ± SD (*n* = 3). Values within a column followed by different small letters are significantly different (*p* < 0.05). RDS—rapidly digestible Starch, AUC—area under curve; CP—control pasta, P5–P20—pasta fortified with 5–20% of fish powder, respectively.

**Table 3:** Protein content, in vitro protein digestibility, and protein availability of pasta fortified with fish powder.

Samples	PC in Raw Pasta (g/100 g Dry Pasta)	PC in cooked Pasta (g/100 g Dry Pasta)	Significance between PC of Uncooked and Cooked Pasta	PD (%)	PA (g/100 g Dry Pasta)
CP	12.20 ± 0.20 ^a^	12.63 ± 0.17 ^a^	*	84.60 ± 0.27 ^a^	10.68 ± 0.12 ^a^
P5	16.67 ± 0.25 ^b^	16.52 ± 0.29 ^b^	*	82.49 ± 0.65 ^b^	13.63 ± 0.31 ^b^
P10	20.08 ± 0.26 ^c^	20.69 ± 0.11 ^c^	*	81.40 ± 0.54 ^b,c^	16.84 ± 0.19 ^c^
P15	25.29 ± 0.14 ^d^	25.15 ± 0.25 ^d^	*	81.52 ± 0.27 ^b,c^	20.50 ± 0.12 ^d^
P20	30.12 ± 0.06 ^e^	29.82 ± 0.29 ^e^	*	80.80 ± 0.37 ^c^	24.09 ± 0.15 ^e^

Mean ± SD (*n* = 3). Values within a column followed by different small letters are significantly different and * indicates not significant (*p* < 0.05). PC—protein content, PD—in vitro protein digestibility, PA—protein availability. CP—control pasta, P5–P20—pasta fortified with 5–20% of fish powder, respectively.

**Table 4:** Total phenolic content and antioxidant activity of fortified pasta subjected to in vitro digestion.

Sample	TPC (mg GAE/g Sample)		ORAC (μmol TE/g Sample)	
	(Gastric Digestion) (0–30 min)	(Pancreatic Digestion) (0–120 min)	(Gastric Digestion) (0–30 min)	(Pancreatic Digestion) (0–120 min)
CP	1.56 ± 0.12 ^c^	2.73 ± 0.08 ^a^	4.39 ± 0.42 ^d^	68.97 ± 4.00 ^d^
P5	1.92 ± 0.02 ^b,c^	3.34 ± 0.04 ^b^	11.07 ± 1.75 ^c^	73.38 ± 8.63 ^c,d^
P10	2.04 ± 0.12 ^b^	3.95 ± 0.16 ^c^	12.84 ± 0.86 ^c^	82.65 ± 3.83 ^bc^
P15	2.74 ± 0.15 ^a^	4.47 ± 0.09 ^d^	20.09 ± 0.55 ^b^	91.83 ± 3.98 ^a,b^
P20	2.77 ± 0.25 ^a^	5.23 ± 0.36 ^e^	24.45 ± 1.41 ^a^	99.31 ± 1.97 ^a^

Mean ± SD (*n* = 3). Values within a column followed by different small letters are significantly different (*p* < 0.05). TPC—total phenolic content, ORAC—oxygen radical absorbance capacity, GAE—gallic acid equivalent, TE-Trolox equivalent value. CP—control pasta, P5–P20—pasta fortified with 5–20% of fish powder, respectively.
